# Complementary and alternative medicine use among patients with type 2 diabetes living in the United Arab Emirates

**DOI:** 10.1186/s12906-020-03011-5

**Published:** 2020-07-10

**Authors:** Hadia Radwan, Hayder Hasan, Rena Hamadeh, Mona Hashim, Zeenat AbdulWahid, Mahboobeh Hassanzadeh Gerashi, Marwa Al Hilali, Farah Naja

**Affiliations:** 1 Department of Clinical Nutrition and Dietetics, College of Health Sciences, Research Institute of Medical & Health Sciences (RIMHS), University of Sharjah, Sharjah, United Arab Emirates; 2 Department of Nutrition and Food Sciences, American University of Beirut, Beirut, Lebanon; 3 Endocrinology and Diabetes Department, Al Qassimi Hospital-Ministry of Health and Prevention, Sharjah, United Arab Emirates; 4 Clinical Nutrition Department, Al Qassimi Hospital-Ministry of Health and Prevention, Sharjah, United Arab Emirates

**Keywords:** Complementary and alternative medicine, Type 2 diabetes mellitus, United Arab Emirates, Folk food and herbs, Patient safety, Integration

## Abstract

**Background:**

The use of Complementary and Alternative Medicine (CAM) among Type 2 Diabetes Mellitus (T2DM) patients is increasing to manage the complexities of their condition, enhance their health, and ease complications. The burden of T2DM in the United Arab Emirates (UAE) coupled with the high prevalence of CAM use and its associated risks among patients with T2DM necessitated the investigation of the use of CAM by this patients’ population. The aim of this study is to examine the prevalence, types, and correlates of CAM use among T2DM patients in the UAE.

**Methods:**

Patients with T2DM attending the outpatient clinics in the two governmental hospitals in Dubai and Sharjah, UAE were invited to participate in a cross-sectional survey. Face-to-face interviews were conducted with participants to complete a multi-component questionnaire. The questionnaire comprised of three main sections: demographic data, diabetes-related information, and CAM use details. Data analysis employed descriptive statistics, univariate and multivariate logistic regression to assess the prevalence and correlates of CAM use.

**Results:**

Two hundred forty-four T2DM patients completed the questionnaire (response rate: 80%). A total of 39.3% of participants were CAM users since diagnosis. After adjustment; the logistic regression results showed that CAM use was significantly associated with age, sex, education, employment, and having health insurance. The most commonly used type of CAM by participants were folk foods and herbs followed by spiritual and natural healing and vitamins and minerals supplements. The majority of CAM users were referred or encouraged to use CAM by family (42.7%), friends (25%) or social media (17.7%). Only 13.5% of participants used CAM because it was suggested by health care practitioners. Only 1 in four of CAM users disclosed CAM use to their treating physician.

**Conclusion:**

CAM use among T2DM patients in the UAE is considerably high. Health policy and decision-makers are encouraged to dedicate particular attention to facilitating proper regulation and integration of CAM within conventional medicine to protect the health and wellbeing of patients. A concerted effort by medical schools and public health authorities should be committed to educating health care providers and patients on the safe and effective use of CAM therapies.

## Background

The burden of Type 2 Diabetes Mellitus (T2DM) is increasing globally, most evidently in developing countries that are experiencing industrial and economic development. These countries have shifted to a more sedentary lifestyle, inadequate eating habits, and less physical activity [1]. Diabetes Mellitus is one of the most significant health care problem and challenge due to its high prevalence and its association with several health complications [2]. Worldwide, the total number of people living with T2DM is projected to increase from 171 million in 2000 to 366 million in 2030 [3]; however, according to the International Diabetes Federation (IDF), these estimates were reached early in 2011 [4]. Among 91 countries studied, 5 of the 10 countries with the world’s highest reported rates of T2DM are in the Eastern Mediterranean Region (EMR), namely the United Arab Emirates (UAE, 17.3%), Saudi Arabia (17.7%), Bahrain (16.5%), Kuwait (15.7%), and Oman (12.6%) [5, 6]. The UAE has experienced rapid economic growth and infrastructure development with the discovery of oil and natural gas over the past three decades [7]. This rapid development has prompted major changes in population lifestyle and health outcomes and has led to an increase in the prevalence of risk factors for T2DM, namely obesity and physical inactivity [8]. Consequently, T2DM has become a major public health burden as it imposes a considerable strain on individuals as well as on the health system in the UAE [9].

Conventional medicine for the management of T2DM has been geared towards regulating blood glucose levels with a combination of dietary changes, insulin and/or oral agents’ utilization, healthy body weight maintenance, regular physical activity, and self-monitoring of blood sugar. However, for many patients with T2DM, the achievement of proper glucose control is often difficult because these conventional treatment regimens require considerable dedication, commitment, and behavioral and lifestyle modifications [10]. Furthermore, T2DM imposes a substantial financial burden on individuals with the disease. The annual medical cost associated with T2DM is 98 billion dollars, including direct and indirect medical expenses and loss of productivity [11]. Patients with T2DM are driven to manage the complexities of their condition, enhance their health, and ease complications through the use of complementary and alternative medicine (CAM) [10, 12, 13]. In fact, the prevalence of using CAM therapies among T2DM patients has reached, in certain reports, rates as high as 70% [14–16]. The most frequently used CAM among these patients were nutritional supplements, herbal medicines, nutritional advice, spiritual healing, and relaxation techniques [14].

Studies examining the reasons for which patients with T2DM used CAM showed that the major driving factors were related to the perceived restrictions and apparent limitations of conventional medicine to treat or manage the chronic disease [17]. Furthermore, the growth in CAM use may also be related to general societal changes, which may be interpreted as a part of the ascendancy of patient self-empowerment [18, 19]. As such, patients pursue the use of CAM because they believe it offers them more personal self-sufficiency and control over their healthcare choices [20, 21]. Also, several researchers have found that there are more complex reasons associated with the use of CAM, such as patients’ values, spiritual and religious ideologies and beliefs, or culture [22].

While several comprehensive reviews have addressed the efficacy of certain CAM therapies in the management of T2DM [23–25], many commonly used therapies remain not verified nor proven [26, 27]. Furthermore, a major concern emerges when patients with T2DM opt to replace clinically proven conventional treatments of the disease with CAM modalities, jeopardizing the efficacy of these treatments and hence the control and management of their condition [28]. Another concern is the risk of CAM-drug interactions when oral CAM are used as complements to conventional treatment. Finally, some of these CAM may worsen glycemic control or generate further complications such as toxicities for patients with T2DM [28]. For instance, the chronic overdose administration of Ginseng may cause gastrointestinal, mental, cardiovascular, and hormone disorders [29]. Also, caution was called for when Fenugreek is combined with aspirin, frequently taken by T2DM patients, as it may increase the risk of bleeding [30]. Moreover, many scientific reports have warned against the excessive and chronic intake of garlic and cinnamon for the treatment of T2DM as it may result in significant adverse health outcomes, such as contact irritation, allergic reaction, or gastrointestinal troubles [31, 32].

In the UAE, traditional and complementary medicine is culturally acceptable and widely utilized, predominantly for the belief that CAM is natural, thus safer than conventional medicine [33]. Previous studies have shown that UAE nationals have faith and confidence in herbal remedies, justifying the high prevalence of CAM use in the UAE [34]. As such, herbal CAM has now been incorporated in the National Health Services alongside conventional medicine, and other CAM modalities such as traditional Chinese medicine, chiropractic, and homeopathy have gained a growing interest and are being officially recognized in the UAE [35–37].

The burden of T2DM in the UAE coupled to the high prevalence of CAM use and its associated risks among patients with T2DM necessitated the investigation of the use of CAM by this patients’ population. Accordingly, the aim of this study was to examine the prevalence, types, and correlates of CAM use among T2DM patients in the UAE. The results of this study will provide supporting evidence that could guide decision making at the system, institutional, and individual level regarding CAM use by patients with T2DM in the UAE, and provide an opportunity for future investigations on the effectiveness of these modalities in the management of diabetes.

## Methods

### Study design

A cross-sectional study design was used to assess the prevalence and types of CAM use among T2DM patients in the UAE. The patients were recruited from outpatient clinics in the two governmental hospitals in Dubai and Sharjah, UAE: Al Baraha and Al Qassimi hospitals. Data collection took place between February 2018–December 2019. The protocol of this study was approved by the Research and Ethics Committee (REC) at the University of Sharjah (Ref no: REC-18-03-07-03-S) and the Ministry of Health and Prevention (MOHAP/UAQ.REC/004/2018).

### Study population

The study population consisted of male and female patients with T2DM visiting the outpatient clinics of Al Barah and Al Qassimi hospitals. Inclusion criteria were: 1-diagnosed with T2DM at least a year ago, 2- aged between 18 and 77 years, and 3- conversant in English or Arabic languages. In order to ascertain their disease status, patients were asked if they were told by their treating physician that they have T2DM. Sample size calculations showed that, in order to detect a prevalence of CAM use of 38% with a power of 80% and a margin of error of 6%, a total of 249 subjects are needed [38]. The prevalence of 38% was identified based on a previous study on the prevalence of CAM use among T2DM in Lebanon [39].

### Study protocol

Trained fieldworkers approached patients in the waiting areas of the hospitals’ clinics. For patients who satisfied the inclusion criteria of this study, the fieldworkers introduced the study objectives and invited them to participate. Interested patients were asked to sign a written consent form and were assured that any information they reveal would remain confidential and would be strictly used for research purposes only. Patients were free to decline to answer any questions with which they were not comfortable. Face-to-face interviews were conducted with participating patients in order to collect the data. Interviewers were trained on maintaining an objective attitude and to refrain from using leading questions. The interviews were conducted on different days of the week and various times during the day. Each interview lasted for 15–20 min.

Data was collected using a multi-component questionnaire, consisting of a total of 48 questions. This questionnaire was used in previous studies assessing the use of CAM in different populations [34, 39–42]. It included three sections: demographic data, diabetes-related information, and CAM usage. Demographic information included gender, age group, educational level, marital and employment status, nationality, religion, and household income. The history of diabetes was assessed by the duration of diabetes, frequency of blood glucose monitoring, last glycosylated hemoglobin A1C (HbA1C) level, presence of any diabetes complications, and family history. The reported results for the patients’ last HBA1C were grouped into normal (controlled) < 6.5 and elevated (uncontrolled) > =6.5 [43]. CAM-related information included types of CAM used, the reason for use, and the source of CAM information. Following a literature review, CAM was grouped into three broad categories to account for the potential variety of therapies: biological-based therapies (such as turmeric, garlic, cinnamon, fenugreek, ginger, black seeds, olive oil, etc.), dietary supplements (such as vitamin B_12_ supplements, multivitamins, vitamin B_6_ supplements, omega-3-fatty acids, grape seed capsules, olive leaf capsules, etc.) and alternative therapies (*Hujama* or wet cupping, Ayurveda medicine, naturopathy, etc.). The questionnaire was originally written in English and was later translated into Arabic. In order to ensure the parallel form reliability, the English version of the questionnaire was translated into Arabic and then back-translated to English. The original and back-translated English versions of the questionnaire were later compared to ensure accuracy in capturing the correct meaning. A copy of the questionnaire is available in Additional file 1.

The questionnaire was piloted on 10 T2DM patients to establish face validity and ensure the clarity of the questions. The results of the pilot study were excluded from the study analysis.

### Statistical analysis

Following collection, the data was entered into Statistical Package for the Social Science (SPSS) software version 24 for Windows. Descriptive analysis was conducted for each of the questions entered in order to validate data entry and detect outliers. In addition, a few cross checks among the questions were preformed to ensure the validity of the data. To describe the characteristics of the participants, categorical variables were presented as frequencies and their corresponding proportions while continuous variables were presented as means ± SD were presented. The main outcome variable in the analysis was CAM use. The latter referred to using CAM at least once since diagnosis with T2DM. In order to determine the differences in characteristics between participants who have used CAM and those who did not, Chi-square (categorical variables) and independent t-tests (continuous variables) were used. Furthermore, the Univariate and multivariate logistic regression analyses were performed to examine determinants of CAM use in the study population. For a variable to be included in the multiple regression model, it had to be significantly associated with the main outcome (CAM use) in the univariate analysis. Age and sex were also included in the multivariate model.. Odds ratios and their respective 95% confidence intervals were calculated. Statistical significance was set at a *p*-value ≤0.05.

## Results

Out of 305 patients approached, a total of 244 patients agreed to take part in this study (response rate 80%). The main reasons for refusal to participate were lack of interest in the study objectives and time/schedule restrictions. Table 1 shows the socio-demographic and disease-related characteristics of the study participants. The average age was 55.8 ± 12.5 years and the females were more than males (64.8% vs. 35.2%). The Emirati patients represented 46.3 and 96.7% of the participants were Muslims. The vast majority (91.7%) were married and only 13.1% had higher education certificates, whereas 40.6% had secondary education and 46.3% were either illiterate or had primary education. Most of the participants were unemployed (62.3%) and only 16.4% were earning more than Arab Emirates Dirham (AED) 10,000 per month (≈United States Dollar (USD) 2700), while 43.9 and 39.8% had a monthly income of AED 5000–10,000 and < 5000 respectively. The majority had health insurance (73%) and T2DM for more than 10 years (61.9%). About half (50.4%) of the patients managed their blood sugar by diet only, while 31.3 and 18.3% were on oral medications and insulin respectively. Data about HbA1c was available only from 70 patients and illustrated that 50% (35/70) of the patients had controlled and 50% (35/70) had uncontrolled HbA1c and among those who responded (210), 88.1% reported family history of T2DM. Almost 50% of the patients stated that they have diabetes complications and 88.1% adhered to doctor’s recommendations.

**Table 1:** Socio-demographic and disease-related characteristics of the study population and their association with CAM use (*n* = 244)^a^

Characteristics	Overall	CAM users	CAM non-users	*p*-value^+^	OR (95%,CI) ^b^
Age (*n* = 244)	55.8 ± 12.5	56.9 ± 13.3	55.1 ± 12.0	0.29	1.01 (0.99–1.03)
Sex (*n* = 244)					
Male	86 (35.2)	32 (33.3)	54 (36.5)		1
Female	158 (64.8)	64 (66.7)	94 (63.5)	0.62	1.15 (0.67–1.97)
Nationality (*n* = 244)					
Emirati	113 (46.3)	43 (44.8)	70 (47.3)		1
Non-Emirati	131 (53.7)	53 (55.2)	78 (52.7)	0.70	1.11 (0.66–1.85)
Religion (*n* = 244)					
Muslim	236 (96.7)	94 (97.9)	142 (95.9)		1
Non-Muslim	8 (3.3)	2 (2.1)	6 (4.1)	0.41	0.50 (0.10–2.55)
Marital Status (*n* = 244)					
Single	24 (9.8)	7 (7.3)	17 (11.5)		1
Married	213 (87.3)	88 (91.7)	125 (84.5)	0.25	1.71 (0.68–4.30)
Widowed or Divorced	7 (2.9)	1 (1.0)	6 (4.1)	0.44	0.41 (0.04–4.01)
Education (*n* = 244)					
Illiterate/ Primary Education	113 (46.3)	39 (40.6)	74 (50.0)		1
Secondary Education	99 (40.6)	48 (50.0)	51 (34.5)	**0.04**	**1.79 (1.03–3.10)**
Higher Education	32 (13.1)	9 (9.4)	23 (15.5)	0.50	0.749 (0.31–1.76)
Employment (*n* = 244)					
Unemployed	152 (62.3)	54 (56.3)	98 (66.2)		1
Employed	92 (37.7)	42 (43.8)	50 (33.8)	0.12	1.52 (0.90–2.58)
Monthly Income (*n* = 244)					
≤ 5000 AED	97 (39.8)	37 (38.5)	60 (40.5)		1
5000–10,000 AED	107 (43.9)	43 (44.8)	64 (43.2)	0.77	1.09 (0.62–1.91)
> 10,000 AED	40 (16.4)	16 (16.7)	24 (16.2)	0.84	1.08 (0.51–2.30)
Presence of Health Insurance (*n* = 244)					
Uninsured	66 (27.0)	18 (18.8)	48 (32.4)		1
Insured	178 (73.0)	78 (81.3)	100 (67.6)	**0.02**	**2.08 (1.11–3.86)**
Diabetes related characteristics					
Duration of T2DM (*n* = 244)					
≤ 10 years	93 (38.1)	39 (40.6)	54 (36.5)		1
≥ 11 years	151 (61.9)	57 (59.4)	94 (63.5)	0.52	0.84 (0.50–1.42)
Management of Blood Sugar Level (*n* = 240)					
Oral Medication	75 (31.3)	29 (30.9)	46 (31.5)		1
Insulin	44 (18.3)	13 (13.8)	31 (21.2)	0.32	0.67 (0.30–1.48)
Diet	121 (50.4)	52 (55.3)	69 (47.3)	0.55	1.20 (0.66–2.15)
HbA1C (*n* = 70)					
Uncontrolled (≥6.5)	35 (50.0)	19 (57.6)	16 (43.2)		1
Controlled (< 6.5)	35 (50.0)	14 (42.4)	21 (56.8)	0.23	0.56 (0.22–1.45)
Family history of diabetes (*n* = 210)					
No	25 (11.9)	11 (12.9)	14 (11.2)		1
Yes	185 (88.1)	74 (87.1)	111 (88.8)	0.70	0.85 (0.37–1.97)
Presence of diabetes complications (*n* = 243)					
No	117 (48.1)	47 (49.5)	70 (47.3)		1
Yes	126 (51.9)	48 (50.5)	78 (52.7)	0.74	0.92 (0.55–1.54)
Adhere to doctor’s recommendations (*n* = 244)					
No	29 (11.9)	15 (15.6)	14 (9.5)		1
Yes	215 (88.1)	81 (84.4)	134 (90.5)	0.15	0.56 (0.26–1.23)

CAM refers to Complementary and Alternative Medicine; ^a^Except for age (continuous variable) which was described as mean ± SD, all variables (categorical variables) were reported as frequencies and proportions; ^+^*p*-values were derived from independent t test for continuous variables and from Chi square for categorical variables; ^b^ OR (95% CI) refers to Odds Ratios and their corresponding Confidence Intervals, OR which were found to be significant at *p* less than 0.05 were bolded

The majority were females (66.7%: 64/96), and non-Emirati used CAM little more than Emirati (55.2% vs. 44.8%). Those who were married had higher odds of using CAM (OR: 1.7; CI: 0.68–4.30) compared to single or widowed or divorced patients. Patients with secondary education had significantly higher odds of CAM use (OR: 1.79; CI: 1.03–3.10) compared to others. Similarly, employed patients showed higher odds of CAM use (OR: 1.52; CI: 0.9–2.58). Patients who had health insurance used CAM two times more (OR: 2.08; CI: 1.11–3.86) than uninsured patients. Patients with T2DM for more than 10 years (OR: 0.84; CI: 0.50–1.42), on insulin treatment (OR: 0.67; CI: 0.30–1.48), controlled blood sugar (OR: 0.56; CI: 0.22–1.45) and those who were adherent to doctor’s recommendations (OR: 0.56; CI: 0.26–1.23) reported lower odds of using CAM as shown in Table 1.

Table 2 shows the correlates of CAM use using multiple logistic regression. The results of the analysis revealed that CAM use is significantly associated with age (OR: 1.03; 95%CI: 1.01–1.06; *p* = 0.01), female sex (OR: 0.51; 95%CI: 0.26–0.99; *p* = 0.05), holding secondary education degree (OR: 2.01; 95%CI: 1.06–3.82; *p* = 0.03), employment (OR: 2.19; 95%CI: 1.07–4.45; p = 0.03) and presence of health insurance (OR: 2.0; 95%CI: 1.05–3.81; *p* = 0.04). The multiple regression analysis was also carried out, with age as a categorical variable. The results showed that older adults (≥52 years) were more likely to use CAM as compared to younger adults (< 52 years) (OR: 2.33, 95%CI: 1.21–4.47). The cutoff for age (52 years) was selected as the midpoint between the youngest and oldest patients participating in this study (Data not shown).

**Table 2:** Correlates of CAM use using multiple logistic regression (*n* = 244)^a^

Characteristic	OR (95% CI) ^b^	*p*-value
Age	**1.03 (1.01–1.06)**	**0.01**
Sex		
Male	1	
Female	**0.51 (0.26–0.99)**	**0.05**
Education		
Illiterate/ Primary Education	1	
Secondary Education	**2.01 (1.06–3.82)**	**0.03**
Higher Education	0.68 (0.27–1.76)	0.43
Employment		
Unemployed	1	
Employed	**2.19 (1.07–4.45)**	**0.03**
Presence of Health Insurance		
Uninsured	1	
Insured	**2.00 (1.05–3.81)**	**0.04**
Adhere to doctor’s recommendations		
No	1	
Yes	0.47 (0.19–1.16)	0.10

CAM refers to Complementary and Alternative Medicine; ^a^Variables with a *p* value less than 0.2 in the bivariate analyses were included in this regression model; ^b^OR (95% CI) refers to Odds Ratios and their corresponding Confidence Intervals, OR which were found to be significant at *p* less than 0.05 were bolded

Table 3 depicts the prevalence, modes, and characteristics of CAM use among patients with T2DM. About 40% (96/244) of the patients were CAM users among whom 90.5% used CAM in the previous year. Most of the patients used CAM as family beliefs or tradition (42.7%) and 25% was suggested to the patients by friends and only 13.5% used CAM as suggested by a health practitioner. About 40% used CAM two times or more per week and 18.8% used it daily. Belief in the benefits of CAM practices was the main reason behind using CAM (66.3%), while 23.2% used CAM because they were disappointed with the conventional medical therapy and about 10% of the patients used CAM just because it was suggested to them. The majority of the CAM users (80%) expected that using CAM might prevent the progression of disease, 6.3 and 3.2% expected to have relief of symptoms and complete cure respectively. Most of the CAM users (74%) reported no side effects from CAM use while 10.4% said yes. Little more than half (55.2%) of the CAM users indicated that they would recommend CAM to other patients with T2DM, whereas 14.2 and 30.2% stated that they would not and undecided respectively. About 68% of the patients are using CAM and most of them did not consult a doctor before using CAM compared to smaller percentage who did (76% vs. 21.9%). Among those who did not use CAM, the reasons were: 1) I do not want additional burden (48.6%); 2) I have never heard of it (17.6%); 3) I do not believe in it (14.2%); 4) My doctor did not prescribe it (14.2%) and 5) I am afraid of the side effects (5.4%). Despite the relatively large number of non-CAM users (148/244; 60%), 79.1% of them stated that they would consider using CAM in the future.

**Table 3:** Prevalence, modes and characteristics of CAM use among patients with T2DM (*n* = 96)^a^

Prevalence and types of CAM used among diabetic patients	n (%)
Used CAM since diagnosis (*n* = 244)	
No	148 (60.7)
Yes	96 (39.3)
Used CAM in the previous year among CAM users (*n* = 95)	
Yes	86 (90.5)
No	9 (9.5)
CAM related characteristics among CAM users	
CAM choice (*n* = 96)	
Family beliefs, traditions, etc.	41 (42.7)
Friends suggestion	24 (25)
Internet, social media, etc.	17 (17.7)
Health practitioner	13 (13.5)
Not applicable	1 (1.0)
Frequency of CAM use (*n* = 96)	
Once/month or less	24 (25.0)
Once/week	16 (16.7)
2 or more times/week	38 (39.6)
Daily	18 (18.8)
Reasons of CAM use (*n* = 95)	
Belief in the benefits of CAM practices	63 (66.3)
Disappointed with conventional medical therapy	22 (23.2)
Trying it as it was suggested to you	10 (10.5)
What was your expectation when you were using CAM (*n* = 95)	
Prevention of the progression of disease	76 (80)
No expectation	10 (10.5)
Symptoms relief	6 (6.3)
Complete cure of disease	3 (3.2)
Side effects from CAM use (*n* = 96)	
No	71 (74)
Yes	10 (10.4)
Not applicable	8 (8.3)
Not sure	7 (7.3)
Would you recommend CAM to other T2DM patients? (*n* = 96)	
Yes	53 (55.2)
Undecided	29 (30.2)
No	14 (14.6)
Did you consult a doctor before using CAM (*n* = 96)	
No	73 (76)
Yes	21 (21.9)
Not Applicable	2 (2.1)
CAM related characteristics among diabetic non-users	
Reasons for not using CAM (*n* = 148)	
I don’t want additional burden	72 (48.6)
I’ve never heard of it	26 (17.6)
I don’t believe in it	21 (14.2)
My doctor didn’t prescribe it	21 (14.2)
I’m afraid of the side effects	8 (5.4)
Would you consider using in the future (*n* = 148)	
No	117 (79.1)
Yes	31 (20.9)

CAM refers to Complementary and Alternative Medicine; ^a^Numbers in this table refer to frequencies (n) and proportions (%)

**Fig. 1:**
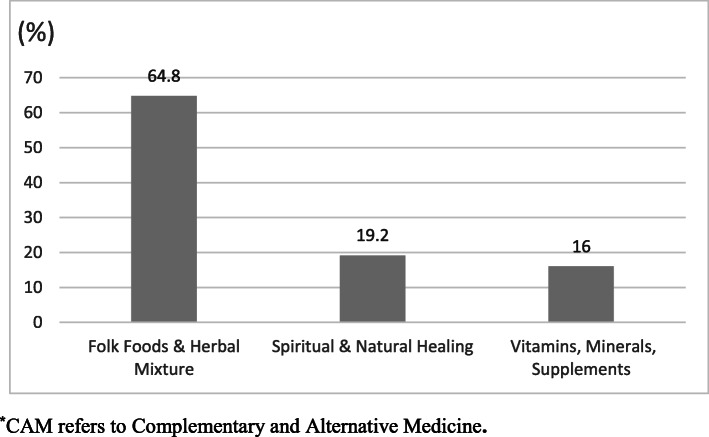
Distribution (%) of various CAM types as used by the study population (*n* = 96)^*^. ^*^CAM refers to Complementary and Alternative Medicine

Figure 1 shows the distribution of various types of CAM as used by the participants. The most commonly used CAM was folk food & herbal mixture (64.8%), followed by spiritual and natural healing (19.2%), and vitamins and minerals supplements (16%). The most frequently used types of folk food and herbal mixtures included; fenugreek, turmeric, cinnamon, garlic, black seeds, ginger, lemon, Arabic gum, bitter gourd, olive oil, onion, coriander, cumin oil, green tea, anise seed, and thyme. While cupping, Ayurveda and naturopathy were the most reported types of natural and spiritual healing techniques used by the participants, multivitamins, vitamin B12, B-complex vitamins, vitamin B6, and omega 3 were predominantly used as supplements by CAM users in this study. All CAM types used by the participants are further illustrated in Additional file 2.

## Discussion

The study findings revealed that 39.3% of participants use CAM therapies since diagnosis with T2DM. This prevalence rate is comparable to findings from the Middle East region, where studies among patients with T2DM revealed a prevalence rate of 30.1, 38, 41, and 41.7% of CAM use in Saudi Arabia, Lebanon, Turkey, and Egypt, respectively [39, 40, 44, 45]. However, studies around the world that examined the use of CAM by T2DM patients have notably different results ranging from 17 to 72.8% [14]. When compared to our study findings, studies in the UK, Germany, and Canada revealed a lower prevalence of CAM use, 17, 18.4, and 25%, respectively [21, 46, 47]. Conversely, studies from Thailand, Taiwan, the USA, Mexico, Korea, and India showed higher prevalence of CAM use with a range between 47.8 and 72.8% [12, 46, 48–54]. These differences in the prevalence of CAM use by region could be explained by the variations in cultural perceptions of CAM use, along with differences in the study design and definition of CAM used by the various studies [14].

The findings of this study showed that the most commonly used type of CAM was folk foods and herbs followed by spiritual and natural healing, and vitamins and mineral supplements. The extensive use of herbal remedies by UAE citizens for both acute and chronic conditions has been previously identified [34]. The Mediterranean region has been distinguished throughout generations with a rich inventory of natural medicinal herbs and has a very rich tradition in the use of medicinal plants for treating various ailments [55]. The UAE is an emerging nation, which is embracing standard treatment approaches to health care but is steeped in traditional healing using folk and herbal medicine [34]. Another reason that could have contributed to the common use of folk and herbal medicine is the fact that these remedies are widely and freely available to UAE residents, with no regulatory control [34]. Among these remedies, the most common herbs used by patients with T2DM in the UAE include fenugreek, turmeric, cinnamon, garlic, black seeds, and ginger. The efficacy of these herbs has been experimentally examined and results suggested that they might possess potential hypoglycemic activity [56–60]. Furthermore, other studies proposed that these herbs may be used in conjunction with anti-diabetic drugs to have better therapeutic potential, minimize oral hypoglycemic drug dosage, and serve as an effective supportive therapy in the prevention and management of long term complications of diabetes [61, 62]. However, evidence for the clinical use of these types of herbal remedies is still scarce; therefore, the covert, concurrent, and frequent use of CAM may confound therapeutic strategies with unexpected outcomes and side effects [63]. Of concern is the fact that in our study, the use of CAM was found rather frequent, whereby 90.5% of patients who have even used CAM did so in the previous year and 39.6% use CAM two or more times per week.

In this study, age, sex, education level, employment and having health insurance were all significant predictors of CAM use among T2DM. The positive correlation found between age and CAM use is in line with the literature, whereby older adults reported more frequent use of CAM as compared to younger adults [64–67]. In fact, Egede et al., identified patients aged over 65 years as being three times more likely to use CAM as compared to those aged less than 65 years [68]. With regard to sex and its association with CAM use, the evidence is inconclusive. While our study showed that male patients were the predominant CAM users, findings from other studies suggested that sex is not a significant predictor of CAM use among patients with T2DM [12, 39, 68, 69]. Several published papers have reported widespread and customary CAM use among the educated and wealthy patients [20, 70, 71]. In this study, CAM use was found to be positively correlated with middle education level, employment, and presence of health insurance. Such associations could be confounded by income: CAM use might increase with employment and having health insurance because people who are employed and are insured also tend to have higher incomes in the UAE; they can better afford to use CAM. In support of this proposition is the fact, that in this study, almost half of non-CAM users indicated that the main reason for not using CAM is the avoidance of additional financial burden, further underscoring the association between income and CAM use.

The findings of this study indicated that the majority of CAM users being referred or encouraged to use CAM by family, friends, or social media’s influence. This is a common finding, as other studies have shown that relatives and friends are instrumental in shaping an individual’s decision regarding the purchase of medicine [72]. On the other hand, in this study, a limited number of T2DM patients were referred to use CAM by their health practitioners and only 1 in four patients informed their treating physician about the use of CAM. Such a finding is in accordance with studies carried out in other countries showing that health care practitioners remain minimally engaged in their patients’ decisions regarding the use of CAM [73–75]. Other studies investigated the reasons provided by patients for not disclosing their use to their healthcare provider and their results converged that the following are the main deterrents: the concern about a negative response from the healthcare provider, fear that the practitioner would not continue providing them with healthcare, fear that physicians would discourage CAM use, their perception that the healthcare practitioner did not need to know about CAM, or simply because the healthcare provider did not ask about CAM use [76]. The marginalization of the health care provider calls for interventions to increase the patient’s awareness regarding the possible dangers that may accompany the unsupervised use of folk and herbal medicine with conventional medicine. Furthermore, this finding provides evidence to elicit a more engaging role of physicians in exploring their patients’ self-use of other forms of treatment. For the latter, the integration of CAM within mainstream medical education could provide a first step in ensuring that physicians are well aware of this prevalent medicinal practice and equip them with the needed knowledge to play a proactive role in their patients’ treatment choices [14, 77].

Some limitations are worth mentioning in this study. Although patients were asked to report their personal experience and opinion and were further assured the confidentiality and privacy of their responses, data collection was completed in the waiting rooms of a hospital setting; therefore, Patients might have experienced the social desirability bias and it is possible that their answers are altered to satisfy their health care providers. CAM use among T2DM patients who are not being followed by healthcare providers or have uncontrolled levels of T2DM might be different in terms of prevalence and type of use, as such a different study design should be employed. Accordingly, the external validity of the findings of the current study might only apply to T2DM patients attending diabetic clinics. Despite this potential bias, the findings of our study highlighted a significant CAM use and a marginal role of health care provides. Additionally, data collected from participants recruited from clinics in governmental hospitals might not be typical for all regions in the UAE, especially for those living in smaller rural communities or major cities, where the living styles and approaches to disease and treatments are likely to be different. It remains important to note that the possibility of type I error (false positive) in examining the predictors of CAM use in the study population could not be ruled out. The current findings of this study may be used in future research in order to employ more informed regression modelling techniques and reduce this type of error, such as Allen-Cady modified backward selection method [78].

## Conclusion

This study revealed a high prevalence of CAM use among T2DM patients in the UAE, particularly for folk foods and herbal remedies. Such use has been embedded within cultural practices and family traditions in the country. Furthermore, CAM use was found to be positively associated with older age and belonging to a higher socioeconomic class. The healthcare providers’ role in orienting patients’ use of CAM modalities was marginal, as patients relied on information from family, friends, and social media, and were less likely to disclose CAM use to their healthcare provider. Health policy and decision-makers should dedicate particular attention to facilitating proper regulation and integration of CAM within conventional medicine to protect the health and wellbeing of patients. Furthermore, a concerted effort by medical schools and public health authorities should be committed to educate healthcare providers and patients on the safe and effective use of CAM therapies. To validate the findings of this study, a national scale study assessing the types and modes of CAM use among T2DM patients is recommended.

## Supplementary information

**Additional file 1.** Questionnaire

**Additional file 2.** Folk Food and Herbal Mixture used by T2DM patients in the UAE.

## Data Availability

The datasets used and/or analyzed during the current study are available from the corresponding author on reasonable request.

## Abbreviations

T2DM: Type 2 Diabetes Mellitus
CAM: Complementary and Alternative Medicine
UAE: United Arab Emirates
IDF: International Diabetes Federation
EMR: Eastern Mediterranean Region
REC: Research and Ethics Committee
MOHAP: Ministry of Health and Prevention
SPSS: Statistical Package for the Social Science
AED: Arab Emirates Dirham
USD: United States Dollar
