# Supplementary material for: Complementary and alternative medicine use among patients with type 2 diabetes living in the United Arab Emirates
Source: BMC Complement Med Ther. 2020 Jul 10;20:216. doi: 10.1186/s12906-020-03011-5 (PMC7350641; doi:10.1186/s12906-020-03011-5)
Supplement: Supplementary file 1 — Additional file 1. Questionnaire [file 12906_2020_3011_MOESM1_ESM.docx]

**Prevalence and determinants of use of complementary and alternative medicine (CAM) among Type 2 diabetic patients in the UAE**

**Questionnaire**

**Date/time (dd/mm/yy) ___/___/___; Subject ID: ______________________**

**SECTION A: DEMOGRAPHICS**

1. **Gender**

a. Male b. Female

1. **Age**  ___________ years
2. **Nationality**
   1. Emirati
   2. Arab
   3. Asian
   4. African
   5. Westerner
   6. Other, please specify _______________
3. **Religion**
   1. Muslim
   2. Christian
   3. Hindu
   4. Other, please specify _________________

1. **Marital status**
   1. Single
   2. Married
   3. Widowed
   4. Divorced
2. **Level of education**
   1. Illiterate
   2. Primary education
   3. Secondary education
   4. Higher education (Diploma, University, Bachelors)
3. **Employment status**
   1. Unemployed
   2. Employed
   3. Housewife
   4. Retired
4. **Household income per month**
   1. Less than 2000 AED
   2. 2000-5000 AED
   3. 5000- 10000 AED
   4. More than 10000 AED
5. **Health insurance**
   1. Uninsured
   2. Public insurance
   3. Private insurance

**SECTION B: Disease History**

1. **For how long you have diabetes?**
   1. Less than 5 years
   2. 5 – 10 years
   3. 11- 20 years
   4. More than 20 years
2. **How do you manage your blood sugar? (Select as applicable)**
   1. Oral medication
   2. Insulin
   3. Diet
   4. Other, please specify ______________

1. **How often do you monitor your blood glucose level?**
   1. Daily
   2. Weekly
   3. Monthly
   4. Never
2. **Do you have any family history of diabetes? (Select as applicable)**
   1. Mother
   2. Father
   3. Both parents
   4. Siblings
   5. Grandparents
   6. Other close relatives
   7. None
3. **Do you suffer from any complication of diabetes?**
   1. Yes
   2. No
4. **If yes, which of the following complications do you suffer from? (Select as applicable)**
5. Peripheral Neuropathy (pins and needles or loss of feeling in the hands or feet)
6. Kidney disease
7. Eye disease
8. Heart disease
9. Stroke
10. Peripheral Vascular Disease (Pain in your legs or feet when you walk short distances)
11. None
12. **Do you suffer from any other chronic health condition? (Select as applicable)**
    1. Hypertension
    2. Heart disease
    3. Cancer
    4. Lung disease
    5. Other, please specify: ______________________
    6. None
13. **On a scale of 1-10, how would you rate the control of your diabetes?**

| **1** | **2** | **3** | **4** | **5** | **6** | **7** | **8** | **9** | **10** |
| --- | --- | --- | --- | --- | --- | --- | --- | --- | --- |

**Very bad Moderate Excellent**

1. **What is your last HbA1c? ____________**
2. **Do you adhere to your doctor’s recommendations?**
   1. Yes b.No
3. **How often do you contact/visit your doctor? _________**
4. **What is the main barrier to your adherence to the doctor’s recommendations?**

a. Unaffordable medication

b. Inconvenient scheduling of the medication

c. Intolerance of drug side effects

d. Others, please specify ____________________

e. None

1. **Have you ever been referred to a dietitian since your diagnosis?**
   1. Yes
   2. No
2. **How often do you contact/visit your dietician for dietary advice?**

a. Once a week

b. Once in two weeks

c. Once a month

d. Once a year

e. Other, please specify _______________

f. Not applicable

1. **Generally, do you follow your dietician’s advice and recommendations?**
   1. Yes
   2. No
   3. Sometimes
   4. Not applicable
2. **Do you think that a dietitian can help you guide and modify your eating habits?**
   1. Yes
   2. No

1. **Who do you think can best help you learn carbohydrate counting?**

a. Your doctor

b. Your dietician

c. You can learn it on your own

d. I do not know what carbohydrate counting is

e. Other, please specify ______________

1. **How important is your diet in regulating your blood sugar?**

a. Not at all

b. Slightly

c. Very

1. **How important is weight maintenance or weight loss (in overweight) in the management of diabetes?**

a. Not at all

b. Slightly

c. Very

1. **How important is regular exercise in controlling diabetes?**

a. Not at all

b. Slightly

c. Very

1. **How often do you exercise?**

a. Once a week

b. 2-3 times a week

c. 4-5 times a week

d. 6-7 times a week

e. I don’t exercise at all

1. **How important is controlling blood glucose in preventing diabetes progression?**

a. Not at all

b. Slightly

c. Very

1. **How would you describe your overall physical health?**

a. Poor

b. Fair

c. Good

d. Excellent

1. **What do you think is the main cause of your diabetes? (Select as applicable)**

a. Age

b. Lifestyle

c. Weight

d. Heredity

e. Other, specify ________________

**SECTION C: COMPLEMETARY AND ALTERNATIVE MEDICINE (CAM) USE**

CAM is a group of diverse treatments that are not currently considered to be part of conventional medicine. Some commonly used CAM include **vitamins/minerals or herbal products or different types of relaxation therapies**. CAM therapies may include special foods such as garlic, bitter gourd, aloe vera, etc. and various spices and seeds such as cinnamon, fenugreek, etc.It can also include yoga, meditation, chiropractor, homeopathy, naturopathy, cupping (hijama), vitamin/mineral or dietary supplements (probiotics, fish oil, etc.).**Please mention ANYTHING that you use for the treatment of your diabetes other than your medication or insulin(Such as cinnamon, garlic, herbs, Yoga, exercise, etc….)**

1. **Have you used CAM since you were diagnosed with diabetes?**

a. Yes b. No

1. **Have you used CAM in the previous year?**

a. Yes b. No

1. **What type of CAM therapies/products have you used?**
   - - 1. Vitamins/Minerals , specify________________
       2. Dietary supplements,specify________________
       3. Special foods ,specify________________
       4. Herbal remedies/Herbal preparations ,specify__________________
       5. Mind-body therapy,specify __________________
       6. Spiritual healing,specify ________________
       7. Folk medicine ,specify________________
       8. Other, please specify _________________
       9. Not applicable
2. **Have you asked your doctor about the CAM therapies/products you used?**

a. Yes

b. No

c. Not applicable

1. **How did you learn about CAM?**
   1. Family beliefs, traditions, etc.
   2. Friends’ suggestion
   3. Internet, social media, etc.
   4. Health practitioner
   5. Other, please specify ____________________
   6. Not applicable
2. **How often do you use CAM?**

a. Once a week

b. 2 or more times per week (for at least a month)

c. Once a month

d. Other, please specify ____________________

e. Never

1. **Why do you use CAM?**

a. Disappointed with conventional medical therapy

b. No other alternative

c. Belief in the benefits of complementary and alternative medicine practices

d. Trying it as it was suggested to you

e. Other, please specify ______________________

f. Not applicable

1. **What did you expect when you started using CAM?**
   1. Complete cure of disease
   2. Prevention of the progression of diabetes
   3. No expectations
   4. Other, please specify ______________________
   5. Not applicable
2. **Do you use CAM for any other medical condition or for your general health (other than your diabetes)?**
   1. Weight loss
   2. Providing energy
   3. Other, please specify ______________________
   4. Not applicable
3. **What was your feeling after using CAM? (Select as applicable)**
   - - 1. Strengthening of body
       2. Being in a good psychological condition
       3. Disappearance of several symptoms
       4. Physically worse
       5. Being in a bad psychological condition
       6. Rise of several symptoms
       7. No change
       8. Other, please specify ______________________
       9. Not applicable
4. **Have you suffered from any side effect of CAM?**
   1. Yes
   2. No
   3. Not sure
   4. Not applicable
5. **Are you currently using CAM?**
6. Yes
7. No
8. I have never used CAM
9. **Would you recommend the use of the CAM treatment to other diabetic patients?**
   1. Yes
   2. No
   3. Undecided
   4. I have never used CAM
10. **If you have not used CAM, would you consider using it in the future?**
11. Yes
12. No
13. I have used CAM
14. **Why haven’t you used CAM?**
15. I’ve never heard of it
16. I’m afraid of the side effects
17. I don’t believe in it
18. My doctor didn’t prescribe it
19. I don’t want additional burden
20. Other, please specify_____________
21. I have used CAM

**THANK YOU**
