# Supplementary material for: Complementary and alternative medicine use among patients with type 2 diabetes living in the United Arab Emirates
Source: BMC Complement Med Ther. 2020 Jul 10;20:216. doi: 10.1186/s12906-020-03011-5 (PMC7350641; doi:10.1186/s12906-020-03011-5)
Supplement: Supplementary file 2 — Additional file 2. Folk Food and Herbal Mixture used by T2DM patients in the UAE. [file 12906_2020_3011_MOESM2_ESM.docx]

Appendix I: Folk Food and Herbal Mixture used by T2DM patients in the UAE.

| **Folk Food and Herbal Mixture** | **Latin binomial nomenclature** |
| --- | --- |
| Fenugreek | Trigonella foenum-graecum L. |
| Turmeric | Curcuma longa |
| Herbal mixtures | - |
| Cinnamon | Cinnamomum Zeylanicum |
| Garlic | Allium sativum L. |
| Black seed | Nigella sativa L. |
| Ginger | Zingiber officinale |
| Lemon | Citrus limon (L.) Osbeck |
| Arabic Gum | Acacia senegal (L.) Willd. |
| Bitter Gourd | Momordica charantia L. |
| Olive oil | Olea europaea L. |
| Onion | Allium cepa L. |
| Coriander | Coriandrum sativum L. |
| Cumin oil | Cuminum cyminum |
| Green Tea | Camellia sinensis |
| Anise Seed | Pimpinella anisum |
| Olive leaves | Olea europaea L. |
| Thyme | Thymus vulgaris L. |
| Almond | Prunus dulcis |
| Apple cider Vinegar | Malus pumila Mill |
| Cucumber | Cucumis sativus L. |
| Cumin Seeds | Cuminum cyminum |
| Flax Seed | Linum usitatissimum L. |
| Grapefruit | Citrus × paradisi Macfad. |
| Honey | Apis mellifera L. |
| Rocca | Eruca vesicaria ssp. sativa |
| Aloe vera | Aloe barbadensis miller |
| Cumin | Cuminum cyminum L. |
| Ajwa seed powder | Phoenix dactylifera |
| Amla powder | Phyllanthus emblica L. |
| Bay leaves | Laurus Laurus nobilis L. |
| Beet root | Beta vulgaris L. |
| Frankincense | Boswellia sacra |
| Orange Peel | Citrus Aurantium Dulcis |
| Parsley | Petroselinum crispum (Mill.) Fuss |
| Teucrium | Teucrium chamaedrys. L. |
| Spinach | Spinacia oleracea L. |
| Karkade | Hibiscus sabdariffa L. |
| Jarjeer | Eruca vesicaria ssp. sativa |
| Guava | Psidium guajava L. |
| Blood Orange | Citrus Sinensis 'Sanguinelli |
| Bottle gourd | Lagenaria siceraria |
| Carrot | Daucus carota subsp. sativus |
| Curry Leaves | Murraya koenigii L. |
| Dill seeds | Anethum graveolens L. |
| Green apple juice | Malus domestica |
| Kiwi | Actinidia deliciosa |
| Legumes | Phaseolus vulgaris |
| Lime | Citrus aurantiifolia |
| Lupine | Lupinus perennis |
| Mint | Mentha x piperita L. |
| Olives | Olea europaea L. |
| Pomegranate | Punica granatum L. |
| Radish | Raphanus sativus L. |
| Sage | Salvia officinalis L. |
| Sauf | Foeniculum vulgare Mill. |
| Yerba Mate | Ilex paraguariensis |
| Sesame | Sesamum indicum L. |
| Walnuts | Juglans regia |
| Red Chia Seed | Salvia hispanica |
| Jamun | Syzygium cumini (L.) Skeels |
| Celery | Apium graveolens L. |
| Cardamom | Elettaria cardamomum |
| Chamomile tea | Matricaria chamomilla |
| Auraq Al Tout | Morus L. |
| Al Murrah Plant | Commiphora myrrh |
| Yansoon | Pimpinella anisum L. |
| Maazaher | Citrus sinensis |
| Alma juice | Phyllanthus emblica L. |
